# Anorexia nervosa, conduct disorder, and the juvenile justice system: a case of applying traditional treatment modalities in a non-traditional setting

**DOI:** 10.1186/s13030-021-00227-w

**Published:** 2021-12-18

**Authors:** Miriam L. Robinovitz, Gregg Joseph Montalto, Khalid I. Afzal, Stephanie Lichtor, Sandeep Palepu, Dena Oaklander, Sarah Carollo, Jonathan Tutko, Jennifer E. Wildes

**Affiliations:** 1 University of Chicago, Chicago, USA; 2 Ann & Robert H. Lurie Children’s Hospital, Chicago, USA

**Keywords:** Anorexia, Antisocial, Conduct, Interdisciplinary, Justice, Juvenile, Non-traditional, Personality

## Abstract

**Background:**

Anorexia Nervosa is highly comorbid with depressive, anxiety, and obsessive-compulsive spectrum disorders. However, it has not previously been reported as comorbid with antisocial personality traits, except when substance use disorder is also identified. We present an unusual case of a patient with resistant anorexia nervosa and comorbid conduct disorder. This case was also unique in that the juvenile justice system was involved during treatment.

**Case presentation:**

A 13-year-old female was admitted to our pediatric hospital for the treatment of anorexia nervosa. She had a history of violent behaviors toward family members, often jeopardizing her care. During hospitalization, she physically attacked a physician on her care team shortly before she transitioned to an eating disorders treatment program. She was diagnosed with conduct disorder, and following discharge, she attacked her father in a premeditated act. This led to her entry into the juvenile justice system. While under the custody of the juvenile justice system, she was readmitted to our hospital for further treatment of anorexia nervosa. Our treatment strategy included psychotropics, positive reinforcement, close interdisciplinary coordination among the various hospital teams, and the juvenile justice system. Following discharge from her second hospitalization back to the juvenile detention system, our patient maintained a healthy weight and appeared to show improvements in the cognitive distortions related to her eating disorder.

**Conclusions:**

To our knowledge, this is the first reported successful treatment of an individual with resistant anorexia nervosa and conduct disorder. It was likely a combination of weight gain, psychotropic medications, and the structured milieu provided by the juvenile justice system that led to the effective treatment of our patient. This case illustrates that a non-traditional healthcare setting can be an asset to treatment through persistence and close collaboration across institutions.

## Background

Approximately 10% of adolescent females in the United States have disordered eating, and the lifetime prevalence of anorexia nervosa (AN) is close to 1 % [1, 2]. Psychiatric comorbidities such as anxiety disorders, mood disorders, and obsessive-compulsive disorder complicate eating disorder recovery [3–5]. The existing literature highlights specific personality disorder traits in patients with eating disorders, including borderline and cluster C personality traits. Still, the prevalence among patients with eating disorders is highly variable between studies [6, 7]. Regardless, there does not appear to be a significant association between AN and antisocial personality traits [7] In their study of eating disorder patients incarcerated in a medical prison, Asami et al. found no significant association between eating disorders and antisocial personality traits among those incarcerated for shoplifting.. Jennings et al. found an association of impulsivity, explosive anger, and aggression with bulimia nervosa and binge-eating disorder, but not specifically with AN [8]. Fava et al. noted a higher incidence of impulsive aggression as compared to premeditated aggression among those with eating disorders [9]. Previous studies have explored the development of antisocial personality traits and conduct disorder in pediatric patients with chronic medical illnesses. However, they did not include eating disorders or malnourishment as chronic medical conditions [10, 11]. The seminal Adverse Childhood Experiences (ACE) study looked at the effects of childhood abuse, neglect, and other adverse childhood experiences. As with most psychiatric disorders, conduct and eating disorders are associated with more adverse experiences in childhood [12–15]. Going further, Horesh et al. implied that eating disorders and conduct disorders were also associated with a greater number of adverse childhood experiences than other disorders among hospitalized children [14]. Recent findings in neuropathology demonstrate altered brain reward circuitry in both conditions [16–18]. We report a successful hospital course of an adolescent with resistant AN-restricting type, major depressive disorder, unspecified anxiety disorder, comorbid conduct disorder with callous-unemotional traits, and premeditated aggression. Furthermore, the treatment was initiated in a pediatric medical hospital, followed by a continuation in a non-healthcare setting of the juvenile detention center, making our case unique and not previously reported in the literature.

## Case presentation

**Fig. 1:**
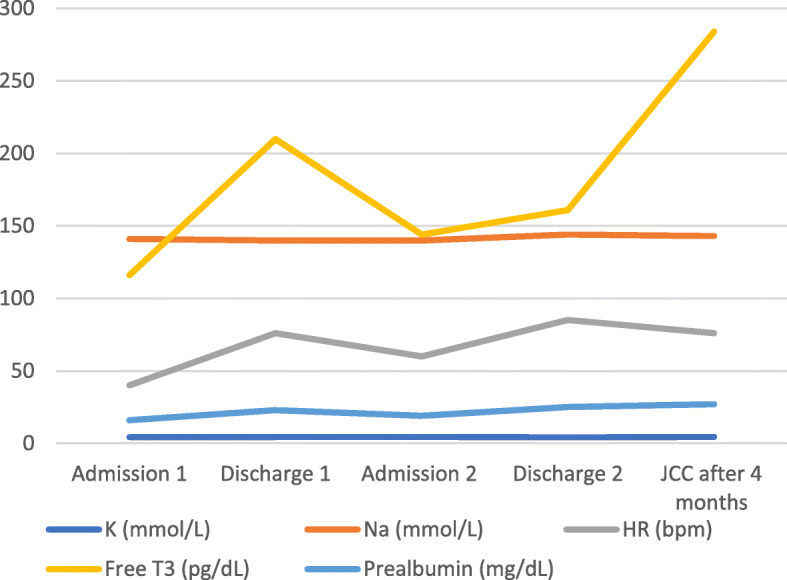
Changes to Potassium, Sodium, Heart Rate, Triiodothyronine and Prealbumin

Our patient was a 13-year-old Caucasian female with a history of anxiety, depression, and anorexia nervosa. She was admitted twice to our hospital. The first admission was in September 2019, when her parents brought her for losing 16 kg over 3–4 months. She was weighing 40 kg (BMI 16.0 kg/m2, 8.7%ile, z = − 1.4) and had sinus bradycardia (HR 30–40 BPM). Table 1 and Fig. 1 show changes in her lab values during her two admissions.

**Table 1:**
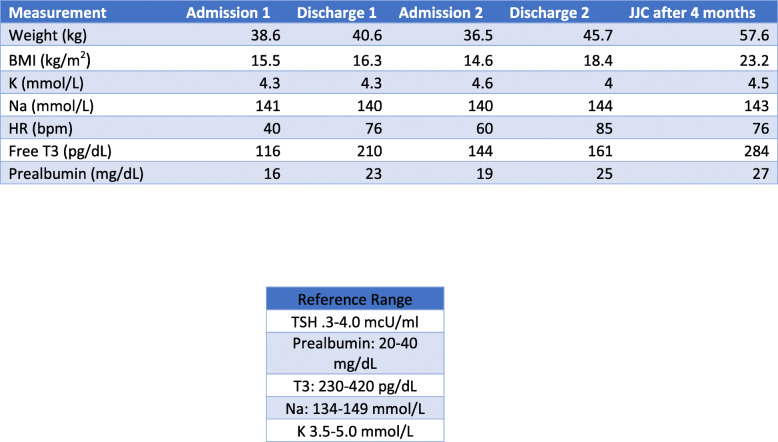
Changes to Pertinent Values Throughout Treatment Course

The patient reported sadness, loneliness, anhedonia, and hopelessness and admitted to restrictive eating behaviors to lose weight. There was no history of purging, bingeing or laxative use. Although emaciated, she minimized the severity of her nutritional status or her preoccupation with her weight and body image. She denied suicidal ideation but desired death rather than pursuing an eating disorder treatment. The patient had compulsive tendencies for academic work and extracurricular pursuits and was highly driven. She had several moves during childhood and reported increased anxiety after a recent move to live with her father following parental divorce. She was starting a new school, as well. We placed her on 1:1 for safety concerns.

The patient started restricting around the age of ten after having a growth spurt at puberty. Following her diagnosis of AN, she received eating disorder treatment at different levels of care, including inpatient, residential, partial hospitalization or Day Program, and intensive outpatient programs in four states. She would become physically aggressive towards staff and family to sabotage her recovery. In one incident, she bit a chunk off the nape of her sister’s neck during a family visit in her residential stay, leading her to be discharged. She admitted to becoming jealous of the sister as she was to go home after the visit while the patient was to remain confined to the unit. She had no remorse about the incident. There was a report where she left the gas stove on to burn the house and kill her mom and her siblings when she was angry. She again had no regrets about her actions. She had history of hurting the family cat. This history of repeated aggression over several years toward other people and animals indicated a possible CD diagnosis. Family history was pertinent for anxiety and mood disorders and AN.

During admission, she hid meals, avoided cardiopulmonary monitoring, and refused nasogastric tube. She would engage in isometric exercises and avoid lying down. She did not take medication for anxiety. As she failed weight restoration in the medical hospital, we referred her to an out-of-state inpatient eating disorder unit for further treatment. Right before the transfer, she jeopardized the transfer by stabbing her pediatrician with a pair of scissors that she hid from the Child Life Services supplies in her room. She intended to hurt the physician for transferring her to the inpatient unit. A room search discovered multiple sharp objects under her mattress, including plastic knives, forks, and medical supplies. For ongoing aggression, she necessitated physical restraints and emergency use of Lorazepam. The patient was formally diagnosed with CD after this incident.

**Fig. 2:**
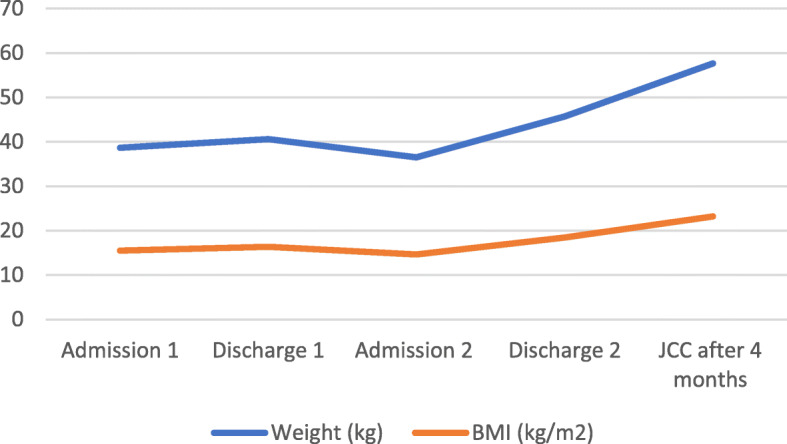
Changes to Weight and BMI. Thoughout Treatment Course

The inpatient eating disorder facility refused to accept the patient after the violence, which led our interdisciplinary team to plan her weight restoration by implementing behavioral expectations and positive reinforcement techniques for meeting daily nutritional needs. Some of the privileges were using the phone, watching TV, or playing video games. We initiated and titrated chlorpromazine 50 mg twice daily for aggression. She gained 2.7 kg in about 6 weeks and her bradycardia resolved (see Fig. 2). Her T3 and prealbumin normalized (see Table 1). After 24 days in the hospital, she was discharged with a plan to start an eating disorder partial hospitalization program.

Following discharge, she participated in the partial hospitalization program for 5 days. On the morning of the sixth day, she entered her father’s bedroom and stabbed him three times with a butcher’s knife. She had purchased the knife the day before to kill her father, blaming him for the eating disorder treatment. She was arrested and remanded to Juvenile Justice Center (JJC), where she started refusing to eat or to take chlorpromazine. She rapidly lost weight and was readmitted for her second admission to our pediatric hospital. On readmission, she weighed 36.5 kg (BMI 14.6 kg/m2, z = − 2.3, 1.0%ile) and was bradycardic and mildly hypothermic with a reduction in T3 and prealbumin. An echocardiogram demonstrated a trace posterior pericardial effusion with normal cardiac function. She was handcuffed and ankle-cuffed to her bed throughout her stay. We switched chlorpromazine to olanzapine (3.5 mg in divided doses), and added fluoxetine (10 mg daily) to address anxiety. She used Lorazepam for anxiety during feedings via NG tube initially. After gaining about 8 kg in about 6 weeks (see Fig. 2), she was discharged back to the JJC, where she continued receiving eating disorder treatment. We suspected that some of the weight gain was related to rehydration. Her pericardial effusion resolved ultimately.

We observed a shift in her statements during weight restoration. For example, “I don’t see how a boy could like me now that I have gained weight” changed to “it does bother me to gain weight, but I try and focus on getting my period back, which makes it easier.” The JJC’s focus on positive reinforcement and reward systems seemingly motivated her to earn additional privileges for desired behaviors. She proudly shared when gaining access to the commissary at JJC, an upgrade in her shoes, a later bedtime, and her ability to spend time with peers in the gym under supervision to prevent over-exercise. JJC staff, many of whom chaperoned her during hospitalization, learned positive reinforcement and practical techniques taught to parents in family-based treatment (FBT) programs and implemented those strategies while she remained incarcerated. Her desire of “never to be hospitalized again” served as a motivation to completing her meals. She had regular follow up with our adolescent medicine team to monitor her progress. As she continued to gain weight, and her anxiety and aggressive impulses decreased. After one visit with our psychiatry team 4 weeks after her discharge, JJC’s psychiatrist took over her psychiatric care and titrated olanzapine to 7.5 mg twice daily and fluoxetine 20 mg daily. Her olanzapine was subsequently decreased to 5 mg twice daily for mild elevation in transaminases that later resolved. Six months after discharge from the hospital, she was still in custody at the JJC, and her nutritional status remained stable. Her menses returned. After complaining of weight gain as an adverse effect of psychotropics, she discontinued olanzapine and later on fluoxetine. However, she maintained her body weight after stopping the medications (see Fig. 2). She has remained in outpatient treatment; and we anticipate that this will be needed long-term given the severity of her symptoms of CD and AN.

## Discussion and Conclusions

To our knowledge, this is the first reported successful treatment of an individual with resistant anorexia nervosa-restricting type (AN), major depressive disorder, unspecified anxiety disorder, and comorbid conduct disorder. The patient initially responded to the interdisciplinary approach at our non-eating disorder pediatric medical hospital and continued to show improvement in the non-healthcare setting of the Illinois Juvenile Justice Center (JJC) where she was incarcerated for violence. While we cannot generalize our treatment success to all patients with conduct disorder or anorexia nervosa, our experience of treating a patient with the unusual combination of both disorders within the framework of the juvenile justice system revealed interesting aspects of treatment for both disorders.

Prominent traits demonstrated in the literature among individuals with AN include maladaptive perfectionism [19], compulsivity [20], and cognitive inflexibility [21]. Our patient demonstrated perfectionism and compulsivity before the onset of her eating disorder, both in her academic and extracurricular endeavors. Her failure to respond to multiple treatments was also not unusual, as AN often is a chronic disorder, with most studies showing a relapse rate at equal to greater than 25% [22]. However, violence toward others and associated anti-social traits are not commonly associated with AN. Before her incarceration for the charges of stabbing her biological father, our patient sabotaged several attempts of eating disorder treatment by exercising violence against her family and medical staff members. She bit her younger sister’s back, drawing blood, during a family visit at a residential treatment center, leading her to be discharged from the unit. She carefully planned and hid scissors under her mattress and attacked the treating physician’s face, with the intention of “gouging his eye out” during her initial hospitalization at our institution. She intended to prevent the physician from transferring her to a residential center. When explored, our patient provided details of her violent acts in a matter-of-fact manner without empathy. Her only concern about her actions was the effect on future relationships with some of the targets of her violence, but no apparent regret of her violent behavior. While patients with eating disorders may be prone to angry outbursts and aggression, it appears that there is a stronger link of intermittent explosive disorder with bulimia nervosa and binge eating disorder than AN [8].

The patient’s pattern of violating the rights of others by use of bullying and threats, weapons, and physical cruelty of others led to a diagnosis of conduct disorder (CD). Her lack of remorse or guilt, absence of empathy or concern about performance, and shallow met criteria for the specifier of limited prosocial emotions. Conduct disorder with this specifier is sometimes is referred to as conduct disorder with callous, unemotional traits (CD-CU). Some studies have suggested that children with conduct disorder who meet criteria for the CU specifier differ in both the origin and psychopathology from other forms of conduct disorder. For example, Viding et al. [23] suggest that CD-CU may be a more heritable form of conduct disorder. In turn, these children appear to be more receptive to positive reinforcement than children with other forms of CD [24]. There is some debate about whether or not personality disorders remain stable following recovery from eating disorders. This would be an interesting point to observe with our particular patient regarding any anti-social personality traits.

At first the restrictions imposed by involvement in the JJC posed a unique challenge to the treatment team, but eventually there were some surprising benefits to the environment within the JJC. Once initial challenge of JJC involvement was the requirement that the patient remain shackled to her bed, Being shackled prevented her from engaging in vigorous exercise; however, the physical restriction became a barrier for her to engage with the interdisciplinary team. Our music therapist and Child Life staff modified their strategies to accommodate the restrictions imposed due to her dangerous behavior. There was also a concern about a breach of confidentiality within existing protocols for detained individuals during the CAP consultations due to the presence of the JJC advocates in the patient room. After deliberate efforts, the JJC modified the protocol to allow CAP consultations in private while the JJC advocates waited outside the room. This privacy encouraged the patient to explore the motivation behind her actions, and her disclosures provided diagnostic clarity regarding conduct disorder traits. Along with the challenges in treating our patient within the JJC, there were some surprising benefits. Perhaps the most significant advantage of incarceration was the prevention of avoidance behaviors. Due to the heavily supervised and highly structured environment of the JJC, our patient was forced to engage in treatment. Without a means to withdraw, our patient’s attention shifted to small goals. First, it was to never again be admitted to the hospital, which she found to be more restrictive than the JJC. During her rehospitalization at our institution, she showed motivation to restore her nutrition in order to achieve a discharge from the hospital back to the JJC. In addition, our patient was very responsive to earning small privileges at the JJC. Her response to positive reinforcement also extended to her response to nutrition restoration in surprising ways. For instance, when our patient earned commissary privileges at JJC, she selected calorie-dense foods as her reward. This was an unexpected benefit of the reward structure provided by the JJC. Research has demonstrated that reward circuitry appears to be altered in patients with eating disorders. For instance, in patients with AN, starvation activates the brain’s reward centers [16]. It is possible that future treatments could harness the altered reward systems in anorexia nervosa. For example, Haynos et al. has proposed adapting a cognitive-behavioral intervention used in depression and anxiety that targets reward deficits to specifically promote non-weight loss related reward responses from patients with AN [25]. This may be a helpful target of therapy given that, unlike with healthy controls, high-calorie food does not activate reward centers in individuals with AN, although low-calorie food does [26]. Because of this, our patient’s response to high-calorie foods is surprising in this context, but she may have viewed these foods as highly prized rewards in the JJC economy since adolescents with AN maintain normal reward circuitry when responding to monetized tasks [26]. Future treatments for eating disorders treatments could similarly incorporate a reward system, and there is at least one instance in the literature suggesting the success of this treatment strategy in patients with AN [27]. Interestingly, there is evidence that children with CD-CU also respond well to positive reinforcement rather than punishment, which may further explain our patient’s overall response to rewards, including to various foods.

Being a restrictive environment, the JJC did not allow access to mirrors. The absence of environmental reminders of the patient’s body image likely had a positive effect on her weight restoration. This observation was evident by the report that her mood seemed to change after she saw an image of herself while speaking over a Zoom video call to her family for the first time in months. The video call coincided with her decision to discontinue psychotropic medication due to her perception of fat redistribution. It has long been established that AN is associated with a disturbance in body image, and the mirrorless environment of the JJC likely protected our patient from her distorted self-perception [28, 29].

There are several limitations to our case report. For one, we cannot say whether our patient’s experience can be generalizable for other patients with similar presentations. In addition, we have limited information regarding our patient’s prehospitalization history, so it is difficult to determine what factors may have contributed to her unusual presentation and if the eating disorder and resulting malnutrition contributed to our patient’s aggressive traits. Similarly, while CD can progress to antisocial personality disorder (ASPD), we do not have the necessary follow up to determine what core features of CD, if any, will persist or lead to ASPD in our patient. In addition, we cannot objectively state how these traits may have changed over the course of hospitalization as we do not routinely use objective assessments for inpatient eating disorder admissions We are not certain which specific interventions led to treatment success. It is unclear whether the substantial decrease in our patient’s disordered thoughts related to her AN were due to weight gain, psychotropic medications, or the new milieu at the detention center, and it was likely a combination of all of these factors that led to an effective treatment. We also wonder if our patient benefited from the focus on positive reinforcement, because of her eating disorder-related circuitry, and not just because of underlying conduct disorder. Further research is warranted into treatments of eating disorders that tap into the specific reward circuitry, utilization of non-healthcare restrictive settings, and personality variations in recovery. Our case was not unique in that we faced various obstacles to treatment. This case illustrates that a non-traditional healthcare setting can be an asset to treating resistant AN, and sometimes, through close collaboration and persistence, it can provide invaluable resources benefiting hard-to-treat patients.

## Data Availability

Not Applicable.

## Abbreviations

AN: Anorexia nervosa
ACE: Adverse childhood experiences
CAP: Child and adolescent psychiatry
CD-CU: Conduct disorder with callous, unemotional traits
FBT: Family-based treatment
JJC: Juvenile justice center
